# Identification of Novel Nexilin Splice Variants in Mouse and Human Tissues

**DOI:** 10.3390/cells13232018

**Published:** 2024-12-06

**Authors:** Paul Jung, André Fiedelak, Celina Dreeßen, Otmar Huber, Juliane Reiche

**Affiliations:** Institute of Biochemistry II, Jena University Hospital, Friedrich Schiller University Jena, Nonnenplan 2-4, 07743 Jena, Germany; paul.jung@med.uni-jena.de (P.J.); andre.fiedelak@med.uni-jena.de (A.F.); celina.dreessen@uni-jena.de (C.D.); otmar.huber@med.uni-jena.de (O.H.)

**Keywords:** nexilin, heart maturation, splicing, isoform, F-actin, T-tubule, epithelium

## Abstract

There is no doubt that the proper development of the heart is important for its correct function, in addition, maturation processes of the heart are crucial as well. The actin-binding protein nexilin seems to take over central roles in the latter processes, as nexilin-deficient mice are phenotypically inconspicuous at birth but die within short time thereafter. Recently, it has been proposed that nexilin plays a role in the formation and function of transverse tubules (T-tubules), which are essential for excitation-contraction coupling in the hearts of mature animals. Although it has long been known that nexilin is subjected to alternative splicing, a molecular characterization of the respective isoforms is not yet available. Here, we describe novel nexilin splice variants and analyze their expression in tissues of mice and humans. Interestingly, nexilin isoforms segregate to myocyte- and epithelial-specific isoforms. Moreover, heart-specific isoforms of nexilin localize differently between atria and ventricles and are also expressed in the endothelial cells of blood vessels. Further, we narrowed down the critical exons in the actin-binding domains 1 and 2 (ABD1/2), and observed different self-interaction properties by recombinant protein interaction studies. Our results emphasize the diverse tissue and subcellular distribution of the individual nexilin isoforms and point to the importance of taking a closer look at the particular nexilin isoforms investigated.

## 1. Introduction

Nexilin was initially discovered as an F-actin-binding protein in rat brain, being most abundant in heart and skeletal muscle [1]. Here, nexilin localizes to Z-discs and serves for the integration and stability of the sarcomere [2]. Several mutations in the nexilin gene (*Nexn*) result in loss of the resistance to mechanical stress in myocytes which leads to dilative or hypertrophic cardiomyopathy [2,3]. Both, nexilin knock-down and overexpression of nexilin mutants in zebrafish led to perturbed cardiac Z-disc stability and dilated cardiomyopathy (DCM). The loss of nexilin in the skeletal muscle did not result in destabilization of Z-discs unless muscle contraction was forced electrically [2]. On the contrary, in a gene-deficient mouse model no morphological abnormalities in the heart were observed at birth. But shortly after birth, homozygous knockout mice developed a progressive dilated cardiomyopathy (DCM) in the absence of notably perturbed Z-discs [4]. At postnatal days 4–6, the relative heart weight was increased more than 2-fold in these mice, whereas the cardiac output was dramatically reduced. Most likely as a consequence of the insufficient heart function, *Nexn*^−/−^ mice died at postnatal days 6–8 (P6–8). Along with these observations, a conventional nexilin-deficient mouse model generated by crossing floxed *Nexn^fl^*^/*fl*^ mice with Sox2-Cre deleter mice also showed no histological abnormalities up to embryonic day 18.5 but rapidly developed severe cardiac enlargement, decreased fractional shortening percentage, and DCM which resulted in early death at day P12 [5]. In line with these findings, heart-specific nexilin knockout mouse models, generated by breeding *Nexn^fl^*^/*fl*^ mice with either cardiomyocyte-specific Xenopus laevis myosin light-chain 2 (XMLC2)-Cre transgenic mice or with cardiomyocyte-specific troponin T2-Cre transgenic mice developed similar phenotypes: well-organized heart architecture at the embryonic stage and rapid progressive DCM with early death before P12. Interestingly, the study by Liu and colleagues suggested a pivotal role for nexilin in transverse (T)-tubule formation and calcium handling rather than in sarcomere stability of cardiomyocytes. Based on their study, the interaction of nexilin with Ca^2+^-handling proteins such as ryanodine receptor 2 (RyR2), the pore-forming subunit Cacna1c, and Junctophilin 2 (Jph2) is a prerequisite for proper T-tubule and dyad formation. In a tamoxifen-induced heart-specific knockout mouse model, the loss of nexilin in adult cardiomyocytes resulted in reduced transversal components of the tubular network, and decreased RyR2 and sarco-endoplasmatic reticulum calcium ATPase (SERCA2) expression. Calsequestrin 1 (Casq1) protein expression was increased in these mice. As a result, the calcium handling was disturbed and the contractility reduced [6].

Around birth, many maturation processes take place in cardiomyocytes, e.g., the effective distance between the sarcolemma and the sarcoplasmic reticulum (SR) is shortened by invaginations of the sarcolemma, the so-called transverse- or T-tubules. This provides close spatial proximity between the L-type calcium channels (LTCC) in the sarcolemma and RyR and Jph2 in the SR, thus enabling rapid signal transduction. Cardiomyocyte maturation is a highly orchestrated process that involves multiple cellular changes: besides structural remodeling, the transformation encompasses, for example, myofibril assembly, changes in calcium handling, alterations in electrophysiology and metabolism, and changes in protein isoform expression [7]. The latter is regulated by alternative splicing events that switch fetal into adult variants. This, for example, applies to titin (Ttn), a giant protein also known as the biological spring of the myocardium. In prenatal mice, longer and more compliant N2BA titin isoforms are predominantly expressed, while in adult mice the ratio changes in favor of the shorter N2B (and also shorter N2BA) variants. This leads to enhanced passive stiffness of the myocardium [8]. Also, the embryonic variant of the mouse myosin heavy chain (Myh7) switches to the adult Myh6 isoform after birth [9,10].

*Nexn* can be alternatively spliced and comprises 14 coding exons in human (XM_005271322; for 13 exon variant: NM_144573) and mouse (NM_001357206). Already in the first description two nexilin isoforms were found in rat tissues: the so-called big variant (b-nexilin) that lacks exon 6 while the small variant (s-nexilin) lacks exon 3. In current gene databases several transcripts have been annotated but none of these transcripts have yet been confirmed by complete transcript sequencing in human tissues. This is of considerable interest because only b-nexilin exhibits F-actin cross-linking activity mediated by two actin-binding sides (ABD1, −2). The ABD1, which comprises exon 2 to 6, is impaired in s-nexilin because exon 3—which is crucial to actin binding—is missing [1]. To date, none of the splice variants have been assigned a specific biological function.

Here, we present a detailed analysis of novel alternatively spliced nexilin transcripts in human and mouse tissues. Furthermore, our data demonstrate that these alternative transcripts segregate in a tissue-specific manner with defined exon clusters. In the heart, these nexilin variants localize to specific regions and also their subcellular localization differs. To our knowledge, this is the first comprehensive description of nexilin splice variants in human and mouse tissues.

## 2. Materials and Methods

### 2.1. Human Samples

Total RNA from human heart, skeletal muscle, urinary bladder, colon, and a frozen human heart tissue block were acquired from Amsbio (Abingdon, UK). All human RNA samples were derived from single healthy donors, except for heart. Here, five different healthy donors served as one pooled sample.

### 2.2. Antibodies

Antibodies targeting nexilin protein were obtained from different companies: Bethyl Laboratories Inc. (Montgomery, TX, USA; A303-806A, rabbit, polyclonal) and Atlas Antibodies (Stockholm, Sweden; HPA011185, rabbit, polyclonal). Antibodies targeting two different epitopes in human nexilin exon 3 were generated by Davids Biotechnologie GmbH (Regensburg, Germany). The peptides “KLGKGDVKDKFEAMQRAREE” (hereafter named KLG) and “REERNQRRSRDEKQRRKE” (hereafter named REE) were synthesized and used to generate antibodies in rabbit and chicken, and to purify antibodies from rabbit serum and yolk sac preparation. Further antibodies used include the following: anti-β-actin, monoclonal anti-Flag (M2) and anti-Myc (9E10) from Sigma-Aldrich (Darmstadt, Germany), anti-GAPDH from Merck-Millipore (Darmstadt, Germany), anti-podocalyxin from R&D Systems (Minneapolis, MN, USA). Horseradish peroxidase (HRP)-labelled secondary antibodies used in Western blot: anti-rabbit-HRP, anti-mouse-HRP were both obtained from Sigma-Aldrich. Antibodies used in immunofluorescence microscopy: anti-rabbit-IgG-Alexa-FITC/594/647 and anti-chicken-IgY-Alexa647 (all Sigma-Aldrich); rabbit-IgG (Thermo-Fisher, Waltham, MA, USA) and chicken-IgY (Davids Biotechnologie GmbH, Germany) served as isotype controls.

### 2.3. RNA Extraction from Mouse Tissues

Adult C57Bl/6 mice (age of two months) of both sexes were decapitated, and the hearts, skeletal muscle (femur), urinary bladder, kidney, colon, and brain were dissected. In order to separate the epithelium from the detrusor muscle, we opened the urinary bladder and stripped off the epithelial tissue from the muscle. The detrusor muscle and the epithelial tissues of five individual bladders were pooled and used in one sample. The RNA of the tissues was extracted using the NucleoSpin RNA extraction kit (Macherey & Nagel, Düren, Germany). In brief, all tissues were lysed and homogenized in RA1 buffer using UltraTurrax (IKA-Werke, Staufen, Germany), and RNA was purified according to manufacturer’s instructions.

### 2.4. Nexilin Transcripts in Human and Mouse Tissues (PCR)

For the analysis of nexilin splice variants 0.1–0.5 ng of total RNA was used in a OneStep-PCR (Luna Universal One-Step RT-qPCR kit, NEB, Frankfurt am Main, Germany) with primers listed in Table 1. According to the manufacturer’s instructions the PCR was performed at 60 °C (30 s; annealing step) and 72 °C (1 min 30 s or 2 min 30 s; extension step) with a total of 40 cycles. After separation of transcripts by agarose gel electrophoresis, PCR products were excised, purified using the NucleoSpin Gel and PCR clean-up kit (Macherey & Nagel), and were sent for sequencing by Macrogen Europe (Amsterdam, The Netherlands).

### 2.5. qRT-PCR on Mouse Tissues

For the quantification of *Nexn* transcripts in mouse tissues and heart regions, C57Bl/6 mice (both sexes; age of two months) were decapitated and whole organs/tissues were collected. Hearts were dissected into atrium, left ventricle and right ventricle. For RNA extraction, tissues were homogenized in lysis buffer RA1 (NucleoSpin RNA, Macherey & Nagel) using UltraTurrax, and column purification was performed according to manufacturer’s instructions. The reverse transcription of RNA (1 µg) into cDNA was performed using High-Capacity cDNA Reverse Transcription kit (Applied Biosystems, Darmstadt, Germany). The qRT-PCR was conducted using GoTaq Sybr Kit (Promega, Walldorf, Germany; for Sybr approach) or Maxima Probe qPCR Mastermix (Applied Biosystems, for Taqman approach to detect Nexn∆3, Nexn∆6, Nexn∆6–8) on a StepOnePlus™ Real-Time PCR System (Applied Biosystems). The data were quantified using the ∆∆Ct method.

### 2.6. Generation of Nexilin Expression Constructs

Nexilin full-length and truncated variants were synthesized in pEX vector by Eurofins Genomics (Ebersberg, Germany) carrying BamHI cleavage sites 5′ of start codon and 3′ of stop codon, and were subsequently cloned into p3xFLAG-CMV10 (Sigma-Aldrich), pCS2+-MT (obtained from Ralph Rupp, LMU Munich, Germany), pQLinkG and pQLinkH vectors (obtained from Konrad Buessow [11]) for further analyses.

### 2.7. Expression and Purification of Recombinant Proteins and Pull-Down Assays

Glutathion-S-transferase (GST) and 7× Histidin (His) fusion proteins (in pQLinkG and pQLinkH plasmid, respectively) were expressed in *E. coli* BL21-DE3 grown in Luria broth medium and induced with 1 mM isopropyl-β-D-1-thiogalactopyranoside. His-tagged proteins were purified using nickel-nitrilotriacetic resin (Ni-NTA). For pull-down (PD) assays, His-nexilin was bound to Ni-NTA resin (1.5 h, 4 °C) in PD buffer (50 mM saccharose, 50 mM NaCl, 10 mM Tris/HCl pH 7.5, 0.5 mM MgCl_2_, 0.5 mM KCl, 50 mM ZnCl_2_, 0.1% [*v*/*v*] Triton X-100). GST or GST-nexilin (bacterial lysate) was incubated with His-nexilin bound to Ni-NTA for 2 h at 4 °C in PD buffer with 20 mM imidazole. Ni-NTA beads were pelleted and washed 4 times in PD buffer with 20 mM imidazole. Finally, the resin was resuspended in 2× Laemmli buffer, and protein complexes were analyzed by SDS-PAGE and Western blotting with GST and nexilin antibodies.

### 2.8. Cell Culture and Immunoprecipitation

Human colon carcinoma cells (Caco2) and human embryonic kidney (HEK-293) cells were used to study nexilin transcript expression and protein interactions. Cells were cultured in Dulbecco’s Modified Eagle’s Medium (DMEM, Sigma, Germany) supplemented with 10% (*v*/*v*) fetal bovine serum and penicillin/streptomycin in a humidified incubator (37 °C, 5% CO_2_). The RNA from Caco2 cells was extracted using the NucleoSpin RNA extraction kit (Macherey & Nagel). For immunoprecipitation of endogenous or overexpressed proteins, HEK-293 cells were seeded at a density of 500,000 cells/6-well. One day after seeding, the cells were transfected with 1 µg DNA mixed with 5 µL polyethylenimine (1 mg/mL). After 48 h, the cells were homogenized in lysis buffer (300 mM sucrose, 150 mM NaCl, 20 mM imidazole, 2 mM MgCl_2_, 0.25% [*v*/*v*] Triton-X100, cOmplete™ protease inhibitor cocktail [Roche, Basel, Switzerland], pH 8.0), centrifuged at 20,000× *g* (10 min, 4 °C), and supernatant was kept for further analysis. For co-immunoprecipitation, 100 µL of the lysate were incubated with 15 µL Protein-A-Sepharose CL4B (GE Healthcare GmbH, Duesseldorf, Germany) pre-bound to 1 µg of Flag antibody under agitation for 16 h at 4 °C. After five washing steps with lysis buffer, precipitated proteins were eluted with 2× Laemmli buffer and analyzed by Western blotting.

### 2.9. Cellular Fractionation

Isolation of cytosol- and membrane-enriched fractions was performed according to Soni et al. [12]. In brief, mouse hearts were homogenized in lysis buffer (20 mM Tris HCl pH 7.4, 1 mM EGTA, 50 mM NaCl, 5 mM NaN_3_, 1 mM PMSF, 50 mM Na_3_VO_4_, protease inhibitor (Complete, EDTA-free, Roche, Basel, Switzerland) using Ultra Turrax, centrifuged at 500× *g* (10 min, 4 °C). The pellet was discarded, and the supernatant was centrifuged at 10,000 × *g* (30 min, 4 °C). The supernatant corresponded to the cytosolic fraction and the pellet dissolved in lysis buffer to the membrane fraction. Equal amounts of both fractions were loaded for Western blotting.

### 2.10. Western Blotting

Proteins, mixed with Laemmli buffer, were separated by SDS-polyacrylamide gel electrophoresis and transferred to polyvinylidene fluoride (PVDF) membranes (Carl Roth, Karlsruhe, Germany). Membranes were incubated with primary antibodies (1 µg/mL) overnight followed by 1 h incubation with HRP-labelled secondary antibody (0.05 µg/mL). Chemiluminescence detection was performed in a G:Box gel documentation system (Syngene, Cambridge, UK).

### 2.11. Immunostaining and Microscopy

C57Bl/6 mice were decapitated, tissues were taken and snap frozen, embedded in Tissue-Tek O.C.T. Compund (Sakura Finetek GmbH, Umkirch, Germany), cryo-sectioned (8–10 µm), and slides were stored at −20 °C. To visualize nexilin, cryo-sections were post-fixed with 4% paraformaldehyde (PFA), blocked with 10% (*v*/*v*) donkey serum in phosphate-buffered saline containing 0.2% (*v*/*v*) Triton-X100 (PBS-T) for 1 h at room-temperature (RT). Primary antibody or isotype control was applied (1:500 in PBS-T, overnight, 4 °C) and detected with secondary antibody (1:1000 in PBS-T, 1 h, RT). Diamidino-phenylindole (DAPI, 1:10,000, Thermo-Fisher) served as nuclear stain. Images were acquired with an AxioImager and AxioCam MRc (ZEISS GmbH, Jena, Germany) and post-processed by using FIJI software (version 1.54i) [13].

### 2.12. Statistical Analysis

The results are presented as mean values ± standard deviation (SD). Data were analyzed using One-Way ANOVA repeated measurements/multiple comparison (Holm–Sidak). Differences with *p* < 0.05 were considered significant.

## 3. Results

### 3.1. Identification of Novel Nexilin Splice Variants in Human and Mouse Tissues

**Figure 1:**
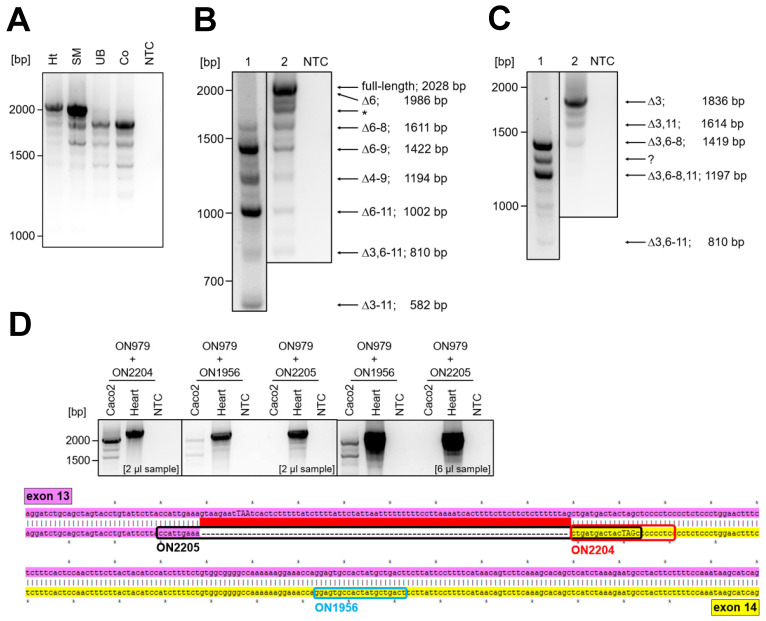

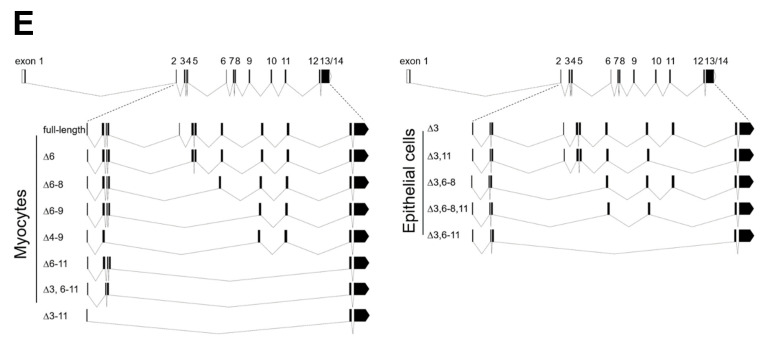
Nexilin transcripts in human tissues. (**A**) Nexilin OneStep-PCR with the use of primers in start (ON979) and stop (ON980) region of protein translation, and subsequent sequencing of the PCR products revealed differences in nexilin transcript pattern between human heart, skeletal muscle, urinary bladder and colon (*n* = 4). (**B**) In human heart, the full-length nexilin and the truncated variant Nexn∆6 are most prominent. The PCR product at ~1800 bp (*) is artificial and arises from fast cooling after PCR (see also Appendix A, Figure A2; *n* = 5). (**C**) In epithelial tissue (colon), the main variant is Nexn∆3. Shorter extension time enabled detection of shorter variants ((**B**,**C**), 1: 1 min 30 s; 2: 2 min 30 s; *n* = 5). The PCR product at ~1300 bp (?) could not be characterized. (**D**) Placing the reverse primer at the exon 13–14 splicing boundary (ON2205, see sequence alignment for primer localization, stop codon in capitals) generated no PCR products in epithelial cells (Caco2) compared to heart sample. However, PCR with reverse primer in the 3′ region of splicing boundary (ON2204, 2 µL sample loaded; ON1956, 6 µL sample loaded) resulted in amplicons in epithelial cells and myocytes. The sequencing of the PCR products confirmed the absence of transcripts alternatively spliced at 3′ end in Caco2 cells. Whereas in the heart both 3′ ends were detected. (**E**) A scheme summarizes the confirmed transcripts in myocytes and epithelial cells. Ht: heart, SM: skeletal muscle, UB: urinary bladder, Co: colon, NTC: no-template control, ON: oligonucleotide.

In 1998, Ohtsuka and colleagues discovered Nexilin as a cytosolic protein in various rat tissues. Additionally, two alternatively spliced variants were described: the so-called b-Nexilin is encoded by exon 1 to 13 but lacks exon 6 whereas s-Nexilin contains exon 6 but lacks exon 3. Despite the confirmation of single exons or parts of nexilin transcripts, no study to date has investigated the expression pattern of complete nexilin transcripts in human tissues. Moreover, the presence of the full-length transcript (encoded by 13 or 14 exons—depending on splicing events at the 3′ end; see below) has not been confirmed by PCR and sequencing approaches. To address this question, we analyzed myocyte samples from human heart and skeletal muscle as well as epithelial samples from human colon and urinary bladder. Primers were designed to target the start and stop regions of the translation to span full transcripts, which could potentially serve for different protein isoforms (ON979—fwd primer in exon 2 which contains the start codon; ON980—rev primer in exon 13—ON980, for localization of primers in human nexilin sequence see Appendix A, Figure A3). Subsequently, all PCR products were sequenced. The sequencing revealed several new nexilin splice variants with varying abundance across the tissues analyzed (Figure 1A).

In human heart and skeletal muscle, the predominant variants were full-length Nexn and Nexn∆6. To allow for a more comprehensive analysis, we varied the PCR extension time from 1 min 30 s to 2 min 30 s and were able to enrich PCR products for predominantly shorter or longer transcripts in heart (Figure 1B, 1 vs. 2) and colon (Figure 1C, 1 vs. 2). Of note, none of the variants detected in human colon or urinary bladder carried exon 3 when we used primers in exon 2 (fwd) and exon 13 (rev). Next, we analyzed the 3′ end of the major nexilin transcripts in myocytes and epithelial cells. For this purpose, the fwd primer localized in exon 2 (translation start, ON979) and the rev primers targeted exon 13/14 boundary (ON2205) or exon 14, which is identical to the 3′ untranslated region in exon 13 (ON2204, ON1956, Figure 1D, scheme). To avoid confusion, we used the nomenclature of exon 14 (as this numbering was described already in mouse) although the generation of the exon 14 is based on an alternative splicing event in exon 13. Sequencing of the PCR products revealed that the 3′ end of epithelial transcripts (colon carcinoma cells—Caco2) is encoded only by 13 exons (Figure 1D). Whereas in the heart, the 3′ ends of the detected variants were encoded by exons 13 and 14. Of note, we also observed transcripts with the 3′ end in exon 14 in Caco2 cells, but only when the fwd primer was placed further downstream of exon 2 (e.g., in exon 10).

Despite the absence of various exons, alternative splicing did not result in a shift in the open reading frame in any of the observed variants. Considering this, and the fact that all detected transcripts carried a translation start and stop suggests that these transcripts represent mature transcripts and can be translated into protein isoforms in the respective tissues (see scheme for transcript summary, Figure 1E).

**Figure A1:**
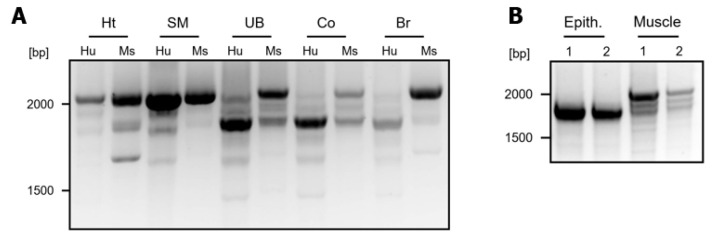
Comparison of nexilin transcripts in human and mouse tissues. (**A**) Human and mouse RNA samples were subjected to nexilin PCR using fwd primer in exon 2 (human: ON979, mouse: ON1014) and rev primers in exon 13 (human: ON980) or exon 14 (mouse: ON1015), following all PCR products were sequenced. (**B**) Urinary bladders derived from ten wildtype mice were dissected into epithelium and detrusor muscle. To gain enough material, five samples each were pooled (1, 2) and analyzed by nexilin PCR (fwd primer: ON1014, rev primer: ON1015). Ht: heart, SM: skeletal muscle, UB: urinary bladder, Co: colon, Br: brain, Hu: human, Ms: mouse, Epith.: epithelium.

When we compared human and mouse tissues we observed a similar transcript pattern for heart, skeletal muscle, urinary bladder, colon, and brain (Appendix A, Figure A1A, human primer: ON979, ON980, mouse primer: ON1014, ON1015; for localization of primers in mouse nexilin sequence see also Appendix A, Figure A4). However, the human RNA samples were generated from biopsies, so the amount of smooth muscle cells (derived from blood vessels) was probably low in these samples. In contrast, mouse RNA was obtained from whole organs which contain blood-vessel derived smooth muscle cells, muscularis (colon) or detrusor (urinary bladder) and thus, quite a number of myocytes. As this may explain the strong signal for myocyte-specific nexilin variants in mouse urinary bladder, colon and brain, we dissected mouse urinary bladders into epithelial and muscle tissue fraction and analyzed both fractions for nexilin expression. As expected, the nexilin variants (primer: ON1014 and ON1015) separated into epithelial and muscle variants (Appendix A, Figure A1B). Sequencing of the PCR products confirmed the tissue-specific distribution of nexilin variants in mouse tissues as we had observed in human tissues.

### 3.2. Nexilin Isoforms Segregate in a Tissue-Specific Manner

**Figure 2:**
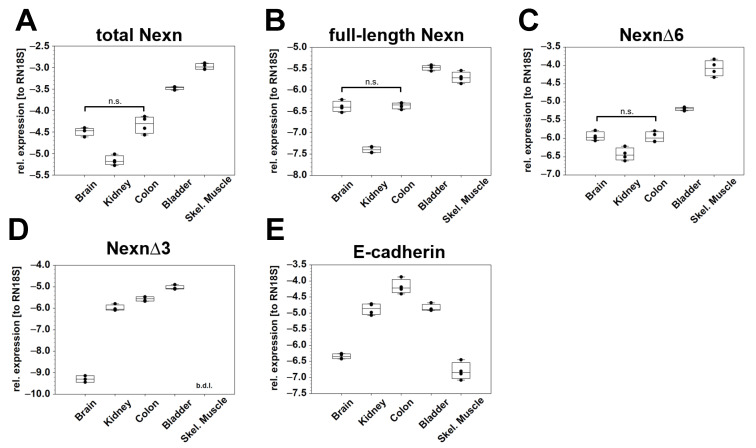
Quantification of nexilin transcripts in mouse tissues. Mouse RNA samples were subjected to qRT-PCR to analyze the expression of different nexilin transcripts in various organs. (**A**) To detect total Nexn, the primers were placed in exon 9 (fwd) and 10 (rev). (**B**) Forward primer in exon 3 and reverse primer in exon 6 enabled quantification of full-length Nexn. (**C**,**D**) Taqman probes spanning exon 5–7 boundary and 2–4 boundary were used to detect Nexn∆6 and Nexn∆3, respectively. (**E**) To quantify the epithelial cells in the analyzed tissues we used primers that target E-cadherin. The relative expression to RN18S (reference gene) is given as base 10 logarithm. For reasons of clarity, we have not plotted the significances in the graphs. There are significant differences (*p* < 0.001) between all groups except for total Nexn, full-length Nexn and Nexn∆6 between brain and colon; and for E-cadherin between kidney and bladder. ANOVA (repeated measures, on ranks, Holm–Sidak), n.s.: not significant, b.d.l.: below detection limit, *n* = 4.

As some of the nexilin isoforms showed higher expression in certain cell types by OneStep-PCR, we aimed to quantify expression levels of total nexilin and nexilin variants using qRT-PCR in mouse brain, kidney, colon, urinary bladder, and skeletal muscle. For total nexilin transcripts (fwd primer in exon 9—ON1707, rev primer in exon 10—ON1765), the skeletal muscle and the urinary bladder exhibited the highest expression. Significantly less total nexilin was found in the brain, colon, and kidney (Figure 2A).

Our results from nexilin OneStep-PCR showed that the exons 3 and 6 are only present together in the full-length nexilin transcript. Therefore, we used a fwd primer in exon 3 (ON1705) and a rev primer in exon 6 (ON1829) to quantify full-length nexilin. The highest expression of full-length nexilin was observed in bladder and skeletal muscle. Lower levels were detected in the brain and colon, and by far the lowest expression level of full-length nexilin was observed in the kidney (Figure 2B). The expression pattern of transcripts, which lack the exon 6 (Nexn∆6) followed the pattern of the total nexilin with high expression in the skeletal muscle and bladder, and lower expression in the brain, colon, and kidney (Figure 2C, fwd primer: ON2059, rev primer: ON2060, Taqman probe: P1). Transcripts which lack exon 3 (Nexn∆3) showed high expression in the kidney, colon and bladder. Low or no Nexn∆3 transcripts were detected in the brain and skeletal muscle, respectively (Figure 2D, fwd primer: ON2196, rev primer: ON2101, Taqman probe: P10). To check if the expression of Nexn∆3 transcripts correlates with the fraction of epithelial cells in the samples, we analyzed the expression of E-cadherin (Cdh1), an adherens junction protein that serves as epithelial cell marker. High expression of E-cadherin was observed in the colon, kidney and bladder, as compared to low expression in the brain and skeletal muscle, which reflects the amount of epithelial cells in these samples (Figure 2E).

### 3.3. Nexilin Expression Differs Between Heart Regions

**Figure 3:**
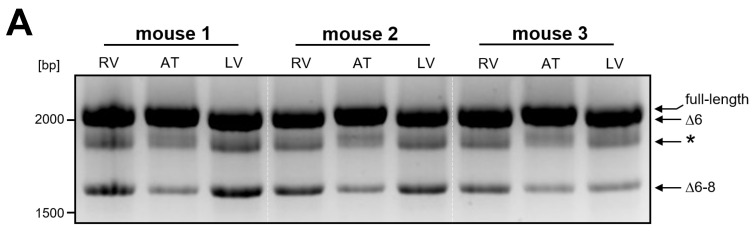

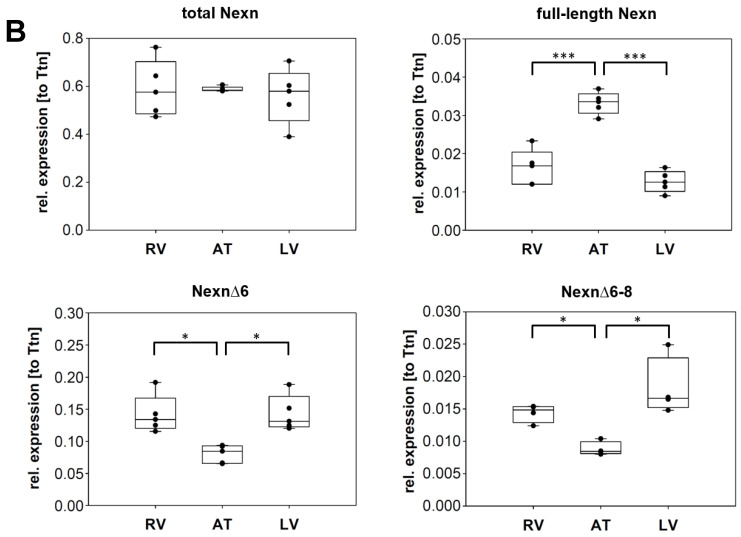
Quantification of nexilin expression in regions of the mouse heart. Analysis of nexilin expression in the atrium and ventricles of the heart was carried out using five wildtype mice. (**A**) Already shown by using OneStep-PCR, faint differences in band intensities were observed between atrium and ventricles. An artificial PCR product (*), which corresponded to the variant Nexn∆6–8, was generated by fast cooling after finished PCR, and could be prevented by reduced cooling speed (see also Appendix A, Figure A2). (**B**) In qRT-PCR we used primer in exon 9/10 (ON1707, ON1765) to detect virtually all nexilin variants (total Nexn). Forward primer in exon 3 (ON1705) and reverse primer in exon 6 (ON1829) served for quantification of full-length nexilin. Taqman probes spanning exon boundary 5–7 (P1) and 5–9 (P3) were used to detect Nexn∆6 (ON2059, ON2060) and Nexn∆6–8 (ON2061, ON2063), respectively. To correct for myocardium, we used titin (Ttn, ON1999, ON2000) as reference. R/LV: right/left ventricle, AT: atrium; *n* = 5 (total, full-length, ∆6); *n* = 4 (∆6–8); *** *p* < 0.001; * *p* < 0.05.

Since the heart is assumed to primarily cause the phenotype of nexilin-deficient mice, we analyzed the expression of the newly identified variants in heart regions in more detail. By PCR (primer: ON1014, ON1015) we could not detect obvious differences in the transcript pattern in the atrium (AT) compared to the right and left ventricles (RV, LV). However, faint differences in band intensities and molecular masses were observed (Figure 3A).

**Figure 4:**
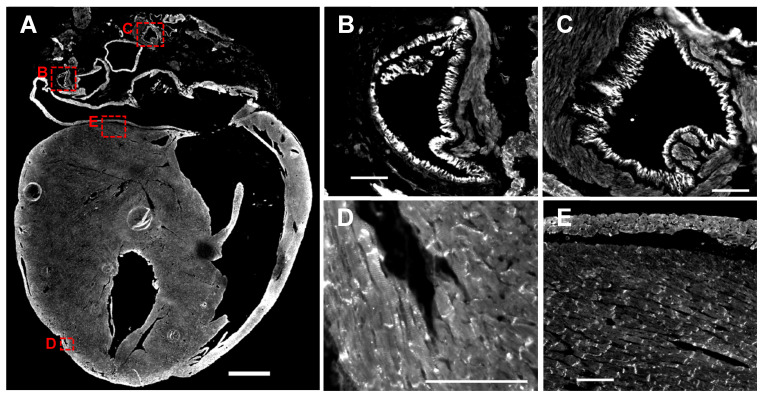

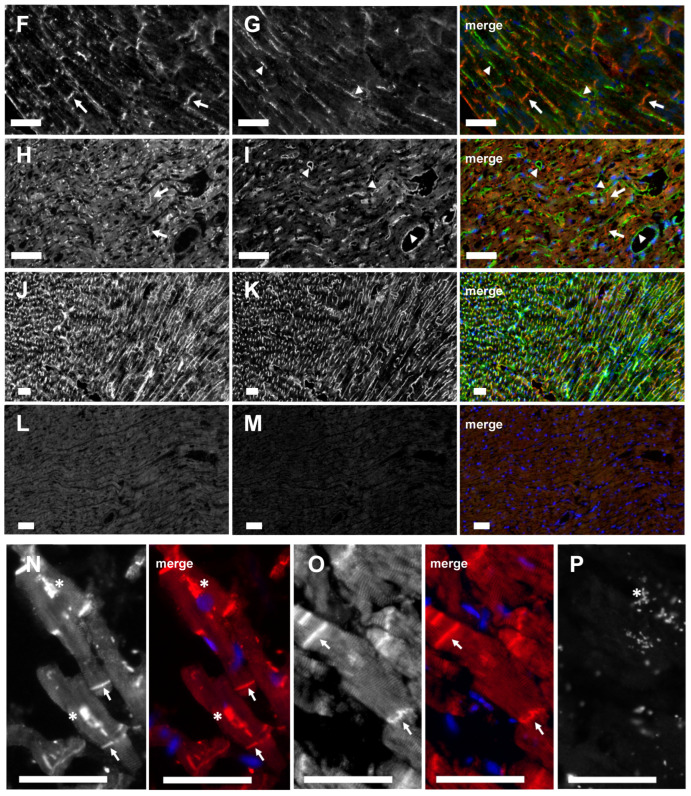
Different localization of nexilin variants in the heart. Representative images of nexilin immunofluorescence staining in mouse (**A**–**M**) and human (**N**–**P**) heart sections. (**A**) Nexilin antibody which was raised against the C-terminus of nexilin (Atlas antibody) revealed signals in endothelial cells of vessels (**B**,**C**), at Z-disc ((**D**), striated pattern) and intercalated discs (**D**,**E**). Co-staining of C-terminal nexilin antibodies ((**F**), Novus antibody; (**H**), Atlas antibody) with antibodies raised against two different peptides of the exon 3 ((**G**), REE peptide; (**I**), KLG peptide; both generated by Davids Biotechnologie GmbH) marked intercalated discs in myocytes ((**F**,**H**), arrows) and structures alongside myocytes ((**G**,**I**), arrow heads). Co-staining of nexilin exon 3 ((**J**), REE peptide) and podocalyxin ((**K**), endothelial marker) revealed similar structures. Isotype control of rabbit IgG (**L**) and chicken IgY (**M**) in mouse heart. Nexilin antibodies targeting the C-terminus ((**N**), Novus antibody; (**O**), Atlas antibody) stained intercalated discs (arrow) and yielded a striated pattern in human myocytes. (**P**) Rabbit IgG control on human heart section. * Autofluorescence of erythrocytes. Scale bar in (**A**): 1 mm, in (**B**–**M**): 100 µm, in (**N**–**P**): 50 µm.

**Figure A2:**
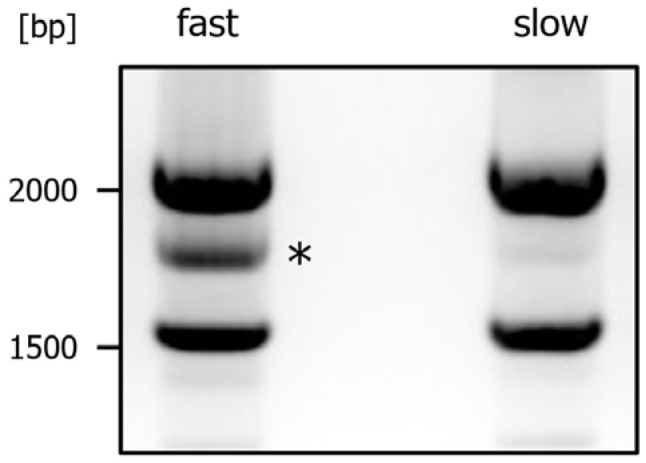
Artificial product formation in nexilin PCR. The PCR on mouse heart RNA revealed three major bands by agarose gel electrophoresis: full-length Nexn and Nexn∆6 migrated in one band at ~2000 bp, Nexn∆6–8 migrated at ~1600 bp and an artificial product run at ~1800 bp (lane “fast”, *). Sequencing of this product revealed identical sequence as to Nexn∆6–8. Lowering the cooling speed down to 1 °C/min after finished PCR reduced the formation of the artificial PCR product (lane “slow”).

**Figure A3:**
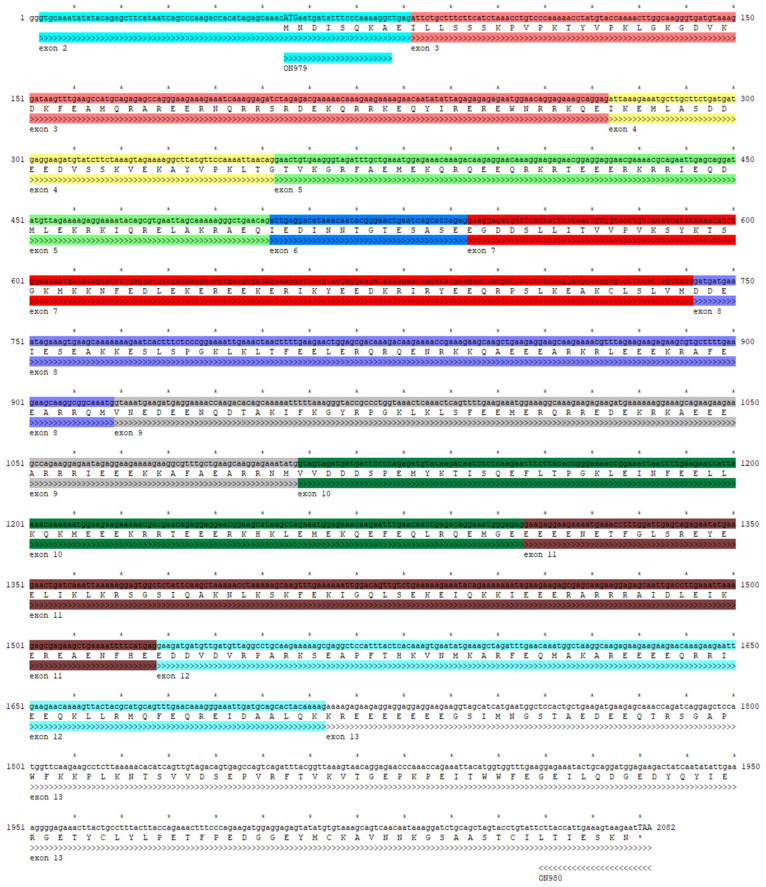
Positioning of primers in human nexilin cDNA [NM_144573]. The exons are highlighted in different colors. The asterisks mark the DNA at intervals of ten nucleotides. The translated amino acid sequence is shown below DNA sequence.

**Figure A4:**
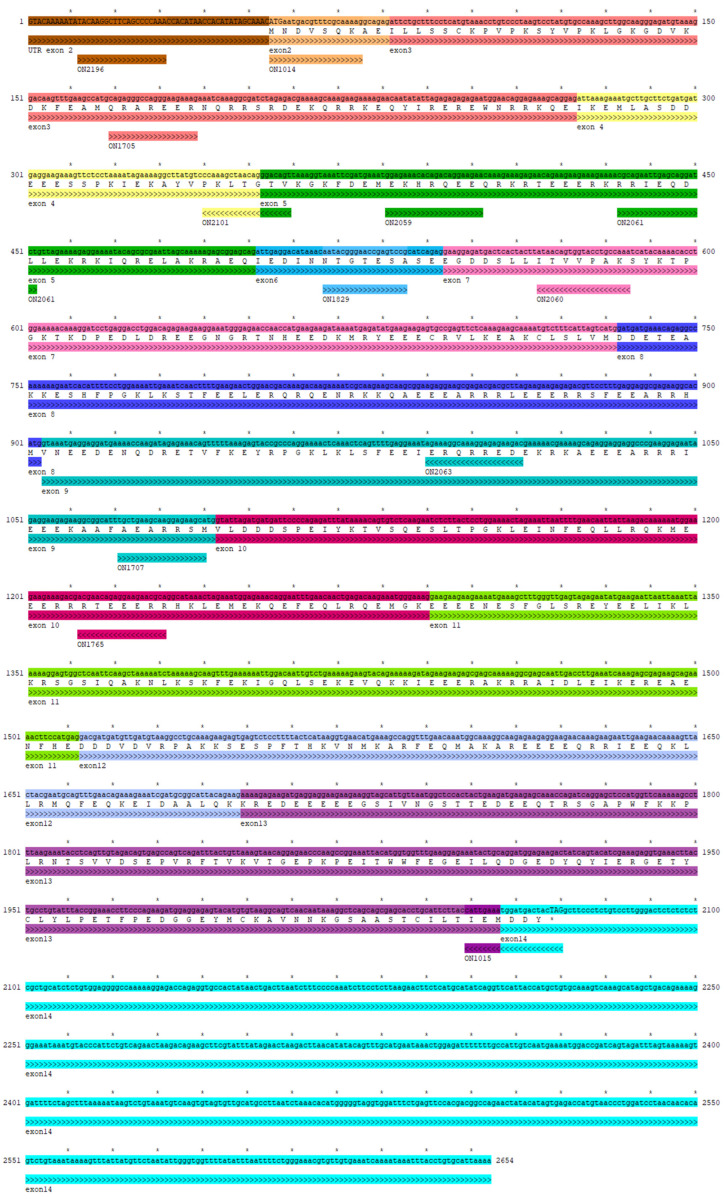
Positioning of primers in mouse nexilin cDNA [NM_001357206]. The exons are highlighted in different colors. The asterisks mark the DNA at intervals of ten nucleotides. The translated amino acid sequence is shown below DNA sequence.

Based on sequencing results, two PCR products migrated as one band at ~2100 bp: Nexn full-length and Nexn∆6. The full-length variant appeared to be more prominent in the atria and/or Nexn∆6 more in the ventricles. In addition, the variant Nexn∆6–8 appeared to be higher expressed in ventricles compared to atria. The Nexn∆6–8 transcript favors the formation of an artificial PCR product that migrates with a slightly higher molecular weight (Figure 3A, *). The sequence of this product was identical to the sequence of Nexn∆6–8 but migrated at a different molecular weight. Formation of this artificial product could be prevented by reducing the cooling speed after completion of the PCR (Appendix A, Figure A2). To quantify the individual transcripts, we performed qRT-PCR. Primers, which target exon 9 and 10 (ON1707, ON1765), and thus do not discriminate between the majority of the transcripts, were used to detect total nexilin. Here, we observed no differences between atria and ventricles (Figure 4B, total Nexn). Expression of full-length Nexn (ON1705, ON1829) was higher in the atria compared to ventricles. In contrast, Nexn∆6 (ON2059, ON2060, P1) and Nexn∆6–8 (ON2061, ON2063, P3) showed higher expression in the ventricles compared to atria. Titin (Ttn, ON1999, ON2000) served as reference to correct for the myocardium.

### 3.4. Nexilin Localization in Mouse and Human Heart

We next determined the localization of nexilin in mouse and human cardiomyocytes. The use of nexilin antibodies, which were raised against different epitopes, enabled the detection of different isoforms. The staining of nexilin on whole mouse heart sections with an antibody which targets an epitope at the C-terminus of nexilin (Atlas antibody) revealed signals at endothelial cells of blood vessels (Figure 4A, for higher magnification see Figure 4B,C), but also at Z-discs (striated pattern in Figure 4D) and at intercalated discs of myocytes (Figure 4D–F,H).

We co-stained nexilin using Atlas and Novus antibody (both raised against the C-terminus) together with antibodies which were raised against different peptides of nexilin exon 3. Exon 3 antibodies stained at the lateral cell membrane or in between cardiomyocytes (REE peptide, Figure 4G; KLG peptide Figure 4I; for complete peptide sequence see materials and methods paragraph). The staining of podocalyxin, which served as an endothelial marker, together with nexilin antibody (REE peptide) revealed co-staining in endothelial cells in mouse heart (Figure 4J,K; rabbit and chicken isotype controls depicted in Figure 4L,M, respectively). Staining of nexilin on human heart sections using antibodies targeting the C-terminus of nexilin showed Z-discs (striated pattern) and intercalated discs (Figure 4N, Novus antibody; Figure 4O, Atlas antibody; rabbit isotype control in Figure 4P).

### 3.5. Nexilin Isoforms Differ in Their Intracellular Localization in Mouse Heart

**Figure 5:**
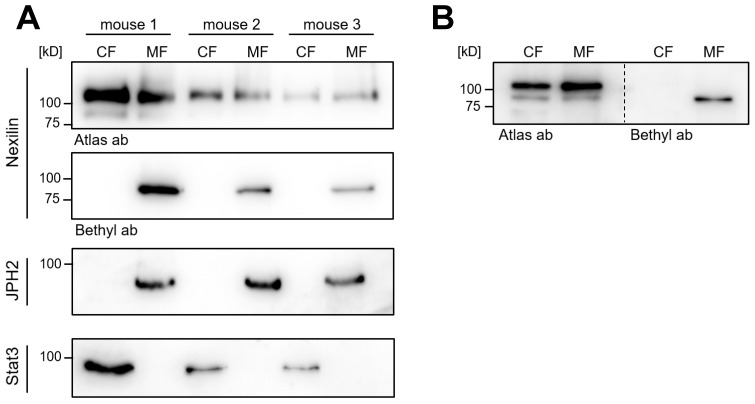
Subcellular fractionation of mouse hearts. Mouse heart tissues were lysed and separated into cytosolic (CF) and membrane (MF) fraction. Nexilin expression in both fractions was analyzed by Western blotting. (**A**) Antibodies, which were raised against different nexilin epitopes, were used to detect nexilin distribution in the fractions (Atlas antibody, Bethyl antibody). Antibodies, which target JPH2 and Stat3, were used to verify cytosolic and membrane enrichment, respectively. (**B**) The migration of the different nexilin isoforms in SDS-PAGE gel was compared by alignment of nexilin blots next to each other.

As shown by Ohtsuka et al., overexpressed b- and s-nexilin localized to the cytosol where they bind to F-actin. Since the presence or absence of exons in the respective isoform could affect binding to interaction partners and thus, could also affect their intracellular localization, we aimed to analyze the cellular segregation of nexilin into the cytosolic or membrane fraction (CF and MF, respectively) in the mouse heart. By subcellular fractionation of three mouse hearts, proteins of the CF and MF were enriched and further analyzed using Western blot. The use of nexilin antibodies, which differ in their target epitopes enabled differentiation between variants. The nexilin antibody from Atlas Antibodies was raised against the C-terminus of nexilin (exon 11–13), which is present in virtually all nexilin isoforms. The Bethyl Laboratories antibody was raised against a peptide sequence encoded by exon 7/8. Variants expressing the C-terminus of nexilin showed no differences in localization between CF and MF (Figure 5A, Atlas ab).

In contrast, nexilin variants, which express the epitope in exon 7/8 were found only in MF (Figure 5A, Bethyl ab). Signal transducer and activator of transcription 3 (STAT3) and junctophilin 2 (JPH2) served as marker proteins for CF and MF, respectively. By aligning the Atlas and Bethyl blot next to each other, the isoforms appear to migrate at different molecular weights (Figure 5B). This would suggest that not all of the nexilin isoforms which express exon 7–8 (Bethyl epitope) also harbor exon 11–13 (Atlas epitope) or vice versa. Alternatively, other differences such as post-translational modifications of amino acids in the respective epitope regions could affect antibody binding to nexilin variants separating to MF and CF. Despite various approaches, we were unable to solve this problem.

### 3.6. Nexilin Exon 3 and Exon 11 Are Crucial for Actin-Binding

**Figure 6:**
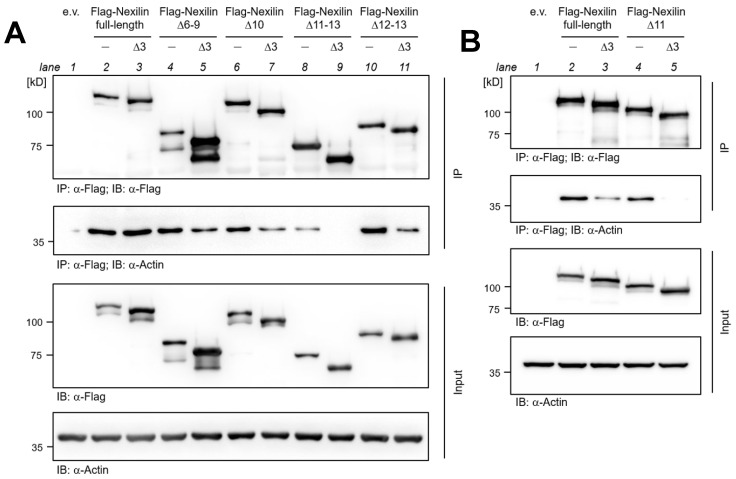
Exon 11 is crucial for actin-binding via ABD2. Representative immunoblots of actin co-precipitated with Flag-nexilin variants. HEK-293 cells were transfected with full-length and the indicated truncated Flag-tagged human nexilin variants. (**A**) Nexilin was precipitated using Flag antibody. Nexilin and co-precipitated actin were analyzed by Western blotting. Deletion of exon 3 impaired binding of acting with nexilin (IP; Actin blot; lane 5, 7, and 11). Removal of the exon 11–13 weakened the interaction between nexilin and actin (lane 8). Additional deletion of the exon 3 abolished the interaction completely (lane 9). Western blot of lysates served as input control. (**B**) In a second setting the exclusive deletion of exon 11 or both, exon 11 and 3, was analyzed. Here, the deletion of exon 3 reduced the actin-binding to nexilin (IP; actin blot; lane 3) and the actin-binding was completely abolished when exon 3 and 11 were deleted (lane 5). e.v.: empty vector; IP: immunoprecipitation; IB: immunoblot; *n* = 5.

Based on the rat nexilin protein, exon 3 was reported to be essential for the nexilin-actin interaction via ABD1, and exons 9, 10, and 11 for binding through ABD2 [1]. Our aim was to narrow down the critical region in the ABD2 in human nexilin. Therefore, truncated, N-terminally Flag-tagged human nexilin variants were transfected into HEK-293 cells and analyzed for their actin-binding ability by co-immunoprecipitation. Deletion of exon 6–9, exon 10, or exon 12–13 had no significant effect on the binding of nexilin to actin (Figure 6A, IP, actin blot, lane 4, 6, and 10 compared to lane 2).

The additional deletion of exon 3 in these variants resulted in impaired co-precipitation of actin, which can be explained by a non-functional ABD1 (lane 5, 7, and 11 compared to lane 3). However, only the nexilin variant that lacks exon 3 and exon 11–13 (Nexn∆3, 11–13) completely lost the ability to bind actin (lane 9). Considering that the nexilin variant, which lacks exons 3, 12, and 13 (Nexn∆3, 12–13) showed nexilin-actin binding (lane 11), the presence of exon 11 seems to be required for a functional ABD2 in human nexilin. Equal expression of transfected Flag-nexilin and endogenous actin was verified by anti-Flag and anti-actin Western blot on cell lysates (Figure 6A, Input). Comparable precipitation of Flag-nexilin was confirmed by anti-Flag Western blot on precipitated samples (IP, Flag blot). In an additional experiment nexilin variants which lacked either exon 3 or exon 11, or both exons were transfected and analyzed for their actin-binding. The absence of exon 3 or 11 led to impaired actin binding compared to full-length nexilin (Figure 6B, IP, actin blot, lane 3 and 4 compared to lane 2). However, actin-binding is completely abolished with the loss of both exons (lane 5). Again, anti-Flag and anti-actin blots confirmed equal expression in transfected cells (Figure 6B, Input), and comparable Flag-nexilin precipitation was confirmed by anti-Flag blot of precipitated protein complexes (Figure 6B, IP, Flag blot).

### 3.7. Direct Interaction of Nexilin Variants

**Figure 7:**
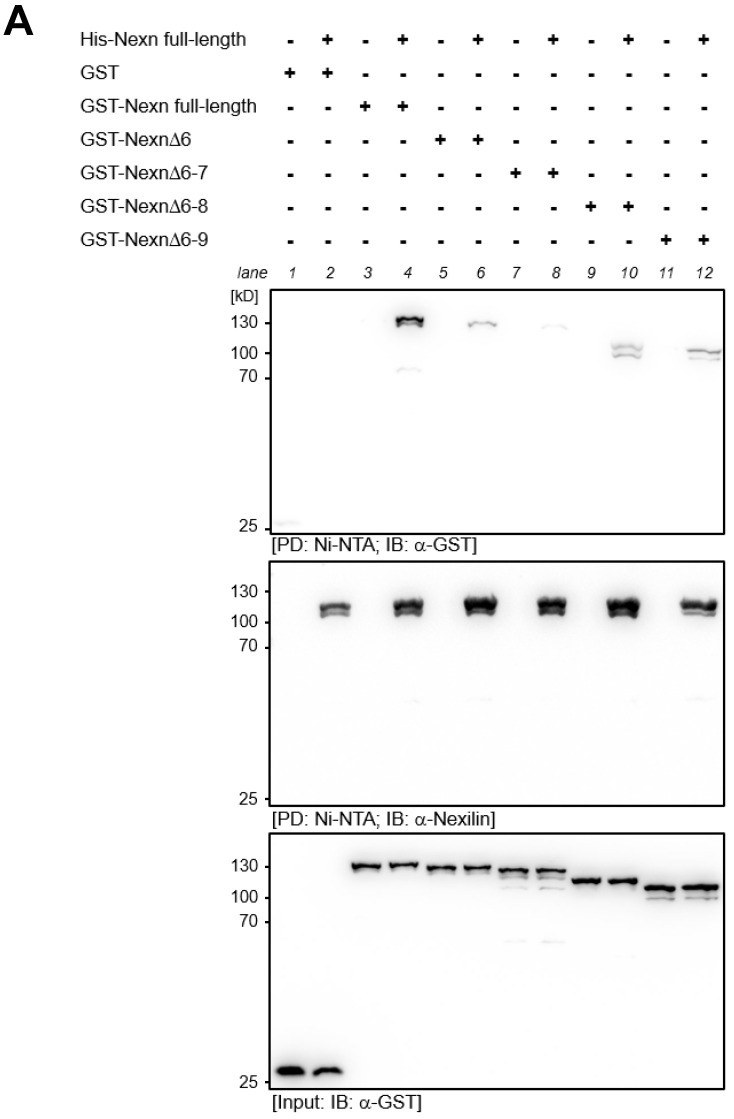

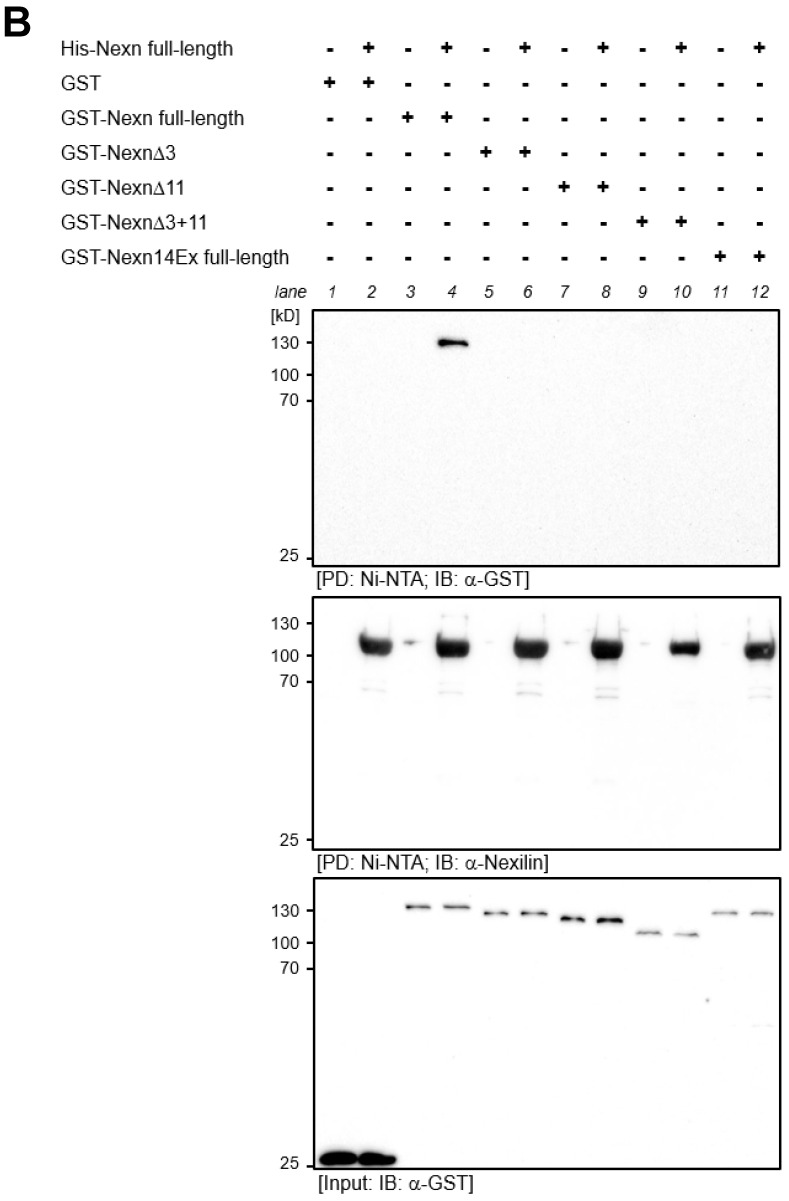
Homomeric interaction of recombinant human nexilin proteins. Full-length and truncated GST- and His-tagged human nexilin variants were recombinantly expressed in *E. coli*. (**A**) His-nexilin was purified on a Ni-NTA column and was used in pull-down assays with GST-nexilin variants. Full-length nexilin forms homomeric complexes (lane 4) but it can also bind to Nexn∆6, Nexn∆6–8, and Nexn∆6–9 proteins although to a lesser extent (lane 6, 10, 12, respectively). Weakest binding was observed between full-length Nexn and Nexn∆6–7 (lane 8). (**B**) In another setting, GST-nexilin variants, which do not harbor the critical exons for the ABD1 (Nexn∆3, lane 6) or for the ABD2 (Nexn∆11, lane 8) or for both (Nexn∆3+11, lane10), showed no interaction with full-length Nexn. Interestingly, the change of the last three amino acids at the C-terminus of full-length Nexn prevented interaction between full-length nexilin proteins (lane 12). PD: pull-down; IB: immunoblot, *n* = 4.

In order to understand the molecular function of nexilin, a homomeric interaction of its variants has to be considered. We examined the formation of homomers using recombinantly expressed His-tagged full-length nexilin and GST-tagged nexilin variants in pull-down assays. In contrast to full-length GST-nexilin, which forms homomers with full-length His-nexilin (Figure 7A, PD, GST blot, lane 4), the deletion of exon 6, 6–7, 6–8 or 6–9 impaired binding between full-length His-nexilin and truncated GST-nexilin proteins (Figure 7A, PD, GST blot, lane 6, 8, 10, 12).

This suggests that the exon 6 is critical for self-association. Interestingly, removal of exon 6–7 resulted in the weakest nexilin–nexilin interaction observed, probably due to changes in the 3D protein structure which impede binding. The nexilin blot after pull-down confirmed equal amounts of His-nexilin bound to the Ni-NTA beads (Figure 7A, PD, Nexilin blot). To verify that equal amounts of GST-nexilin variants were used, input material was collected for Western blot analysis before resin-bound His-nexilin was added (Figure 7A, Input, GST blot). In addition, the deletion of critical exons for either one of the actin-binding domains (Figure 7B, PD, GST blot, lane 6 and 8), or for both (lane 10) prevented the interaction of nexilin proteins. Interestingly, changing the last three amino acids of the C-terminus of nexilin from serine, lysine and asparagine to threonine, aspartate, aspartate, and tyrosine as a consequence of exon 13 or 14 usage impaired self-interaction completely (lane 12).

**Figure 8:**
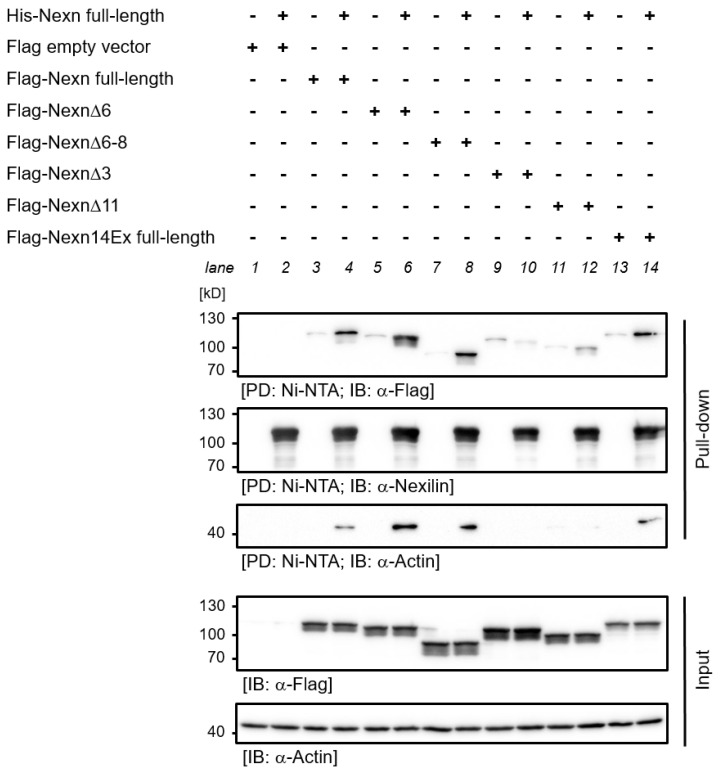
Pull-down of human nexilin protein variants from HEK-293 cells lysates. Full-length His-tagged human nexilin was recombinantly expressed in *E. coli*, purified on a Ni-NTA column and was used in pull-down assays with lysates of HEK-293 cells overexpressing Flag-tagged nexilin variants. Full-length nexilin forms homomeric complexes (PD, Flag blot, lane 4) but it can also bind to Flag-Nexn∆6 (lane 6), Flag-Nexn∆6–8 (lane 8) and to full-length Flag-nexilin with different amino acids at the C-terminus (lane 14). Deletion of exon 3 or exon 11 (lane 10 and 12, respectively) prevented interaction between nexilin variants. Co-precipitation of endogenous actin from HEK-293 cell lysates seemed to be dependent on critical exons in ABD1 (PD, actin blot, lane 10) and in ABD2 (lane 12 compared to lane 4, 6, 8, and 14). Nexilin blot of PD confirmed equal amounts of His-nexilin (PD, Nexilin blot). Flag and actin blot of input material confirmed equal expression of Flag-tagged nexilin and endogenous actin (Input, Flag and actin blot). PD: pull-down; IB: immunoblot, *n* = 5.

We repeated the interaction study with His-tagged nexilin bound to Ni-NTA column and lysates from HEK-293 cells which overexpressed Flag-tagged nexilin variants. Surprisingly, here Flag-tagged Nexn full-length, Nexn∆6 and Nexn∆6–8 bound to full-length His-nexilin (Figure 8, PD, Flag blot, lane 4, 6, 8). As observed above, deletion of exons crucial for actin-binding (exon 3 or 11) resulted in an impaired nexilin–nexilin interaction (lane 10, 12). In this experimental setting we observed an interaction between full-length His-nexilin (C-terminus encoded by exon 13) and Flag-tagged nexilin with a different C-terminus (encoded by exon 14, lane 14), in contrast to the experiments with protein variants expressed in *E. coli*. Interestingly, nexilin–nexilin interaction was only observed when actin was co-precipitated (Figure 8, PD, actin blot). Comparable amounts of Ni-NTA-bound His-nexilin was shown by anti-nexilin blot in pull-down fraction. Equal expression of Flag-tagged nexilin variants and endogenous actin was confirmed by anti-Flag and anti-actin blot (Input).

## 4. Discussion

DCM is characterized by a left ventricular (LV) systolic dysfunction with LV enlargement. Up to now, more than 260 gene loci are associated with DCM, out of these only 19 genes were found to support a single-gene, Mendelian cause of DCM—with *Nexn* being one of these genes [14]. Interestingly, there are several studies describing cardiac pathologies associated with *Nexn* mutations, but none of them analyzed the expression pattern of nexilin splice variants in cardiomyocytes [2,15]. Here, we provide evidence that (i) nexilin is alternatively spliced in the heart and other tissues, (ii) that these splice variants are differently expressed in heart regions, and (iii) that these variants exhibit a different subcellular distribution.

When nexilin was first described in rat tissue, two splice variants were reported, both encoded by 13 exons but lacking either exon 6 or exon 3 [1]. Interestingly, a nexilin variant, which expresses all exons (full-length) was not observed but was proposed in later publications. Even though a considerable number of studies employing human, mouse or zebrafish models have been published in the meantime, to our knowledge no alternatively spliced mature nexilin transcripts were reported to date. In 2014, Yang and colleagues investigated the role of nexilin in the process of cardiac development using an embryonic mouse cell line [16]. By PCR and the usage of different primers they detected three splice variants (Nexn-α, -β, -γ). However, according to the published data, the primers they used targeted exons which were not present in the proposed variants. Moreover, the primers only covered the region between exon 5 and 9. Any information about the adjacent exons or the complete transcripts is missing. To our knowledge, our study provides the first comprehensive panel of alternatively spliced nexilin variants in human and mouse tissues. We confirmed expression of the full-length nexilin transcript in skeletal muscle and in cardiomyocytes and obtained several new variants in cardiac and skeletal muscle by sequencing complete transcripts. We could not detect full-length transcripts (all exons from 2 to 13/14) in non-myocytes suggesting that these isoforms are only be expressed at a low level in these cells compared to the prominent Nexn∆3 variant. An alternative splicing event at the 3′ end resulted in an additional intron of 81 bp and by that shortens exon 13 and adds exon 14 (see scheme in Figure 1D). This leads to a change in the C-terminal amino acid sequence: from polar (p) and positively charged (+) amino acids (serine, lysine, and asparagine; p + p) to polar and negatively charged (−) C-terminus (threonine, aspartate, aspartate, and tyrosine; p − − p) in human. In mice and rats, threonine in the negatively charged C-terminus is substituted by methionine (non-polar). Again, this splicing event seems to be tissue specific. We could not detect transcripts carrying exon 14 in epithelial samples when placing the forward primer in exon 2. In contrast, the majority of the transcripts in the human heart carried the additional exon 14 suggesting an essential function of these last four amino acids predominantly in cardiomyocytes. Interestingly, when using a forward primer binding to more 3′-shifted positions (e.g., exon 10) we also detected transcripts with exon 14 in epithelial cells—unfortunately, without any further information about the complete transcript. Since there are no post-translational modifications of the C-terminal amino acids described so far, such as acetylation or ubiquitination of the lysine or phosphorylation of the serine, threonine or tyrosine (www.phosphosite.org (accessed on 15 August 2024)), it can be assumed that the change in the charge of the C-terminus is necessary, for example, to bind interaction partners. Also, it is conceivable that differences in the amino acid sequence at the C-terminus are sufficient to affect the protein structure even at the N-terminus—as predicted by AlphaFold [www.alphafold.ebi.ac.uk (accessed on 15 August 2024), NP_653174 vs. XP_005271379]. However, feeding AlphaFold with the human full-length nexilin protein sequence (NP_653174, exon 13 variant) unexpectedly resulted in a (quite) different structural model compared to the model predicted by the database-given human full-length nexilin (exon 13 variant, UniProt: Q0ZGT2—which is based on the exact same amino acid sequence).

In a recent study, Vad and colleagues analyzed potential target genes of the splicing regulator RNA binding motif protein 20 (RBM20) in rat atria. Differential transcript usage analysis in a *RBM20*-deficient rat model among others revealed that *Nexn* splice transcripts were differentially regulated in the atria of knockout rats compared to atria of wildtype rats [17]. The observed five variants reflected the total of nexilin transcripts listed in ENSEMBL database [www.ensembl.org]. It would be of interest, if (i) additional nexilin transcripts in general—independent of *RBM20* genotype—were observed, (ii) if RBM20-dependent splicing resulted in the same differential nexilin transcript pattern in the ventricles of these hearts, and (iii) if mutations in *RBM20* lead to altered nexilin splicing. Up to 6% of all genetic cardiomyopathy cases originate from mutations in *RBM20* [18]. It would therefore be interesting if altered nexilin splicing, which favors the expression of certain isoforms (possibly as a consequence of *RBM20* mutations?), can lead to DCM.

The observation that Nexn∆3 is the main variant in epithelial cells sheds some light on the role of the ABD1 in epithelial cells because exon 3 is crucial to actin-binding via ABD1. The presence of two ABDs supports a function of nexilin in cross-linking actin filaments with defined spacing. Actin filaments—together with microtubules and intermediate filaments—constitute the cytoskeleton of cells and by that provide mechanical support, drive morphological changes and enable cell motility. To fulfil these needs, actin filaments are cross-linked in different ways which give rise to their various forms. Networks of branched actin filaments (e.g., cross-linked by spectrin) act primarily on pressure, whereas bundled actin (e.g., cross-linked by fascin) is required when forces act mainly in one direction, as is the case with filopodia. Non-aligned actin filament network (e.g., cross-linked by filamin) withstands forces in multiple directions, and stress fibers (e.g., cross-linked by myosin) generate tension [19]. Among actin cross-linking proteins, small cross-linkers tend to be globular and exhibit more than one actin-binding site (e.g., fascin, fimbrin). Large cross-linkers create more space, generate actin networks and usually have only one actin-binding site. One could speculate that, depending on the specific isoform, nexilin may serve as a cross-linker to promote tight actin filaments or loose actin networks. In myocytes, where the most prominent isoforms (Nexn full-length, Nexn∆6 and Nexn∆6–8) exhibit two ABDs, nexilin could serve as a cross-linker to support bundled actin filaments with different spacing. In this case, the presence of the coiled-coil domain (exon 8/9) could also promote the formation of higher order assemblies. In contrast, most nexilin variants in epithelial cells carry only one functional ABD. Therefore, a cross-linking function of nexilin in loose filament networks or stress fibers seems more likely. Nexilin variants with no functional ABD (lack of exon 3 and 11) have also been observed. Hence, functions independent of actin are also conceivable. Interestingly, in our in vitro binding experiments, we only observed strong homomeric self-interaction of full-length nexilins. Truncated variants exhibited only weak interaction with full-length nexilin—if at all. And surprisingly, even a relatively small variation in the C-terminal amino acid sequence (and therefore altered charge of the C-terminus in exon 13 vs. exon 14 variants) also affected self-interaction of nexilin. However, the presence of ABDs seemed to be crucial for nexilin–nexilin complexes as the presence of potential binding partners (e.g., actin; as observed in pull-down experiments using HEK-293 cell lysates) rescued binding of full-length nexilin to Nexn∆6, Nexn∆6–8 and Nexn14Ex. This was not observed in the absence of exons that have been shown to be critical for actin binding (Nexn∆3, Nexn∆11). Therefore, it seems unlikely that a relevant amount of nexilin–nexilin complexes is formed in epithelial cells, where Nexn∆3 and Nexn∆3,6–8 are the major variants. However, a pull-down assay using HEK-293 cell lysates, as we have applied here, is not a direct-binding assay in the strict sense, nevertheless provides important information.

### Preferential Expression of Nexilin Isoforms in Different Areas of the Heart

Across the heart, nexilin isoforms revealed a distinct expression pattern in different areas. Full-length nexilin was primarily found in atria, truncated nexilins (Nexn∆6, Nexn∆6–8) were prominent in ventricles. The atria function as a reservoir for venous blood and as a contractile chamber to reinforce ventricular filling. As far as the composition of the walls is concerned, both the atrial and the ventricular wall consist of three layers: the outer epicardium, the myocardium and the inner endocardium. Due to the greater force required to pump blood out of the ventricles, the myocardium of the ventricle is thicker than that of the atrium. When we analyzed nexilin expression we normalized to myocardial titin. Therefore, different fractional amounts of myocardial tissue in the samples cannot explain the differences in nexilin expression. However, there are well known differences in the cellular morphology, Ca^2+^ homeostasis and the expression of structural or Ca^2+^-regulatory proteins between atrial and ventricular myocytes. For example, the ratio of surface area to volume is increased in atrial vs. ventricular myocytes. On protein level, the myosin light chain-2 (MLC2), responsible for sarcomere formation, segregates into an atrial (MLC2a) and ventricular (MLC2v) isoform [20]. The Ca^2+^-ATPase SERCA shows higher expression levels in atria than in ventricles, whereas the SERCA inhibitor phospholamban and the calcium-binding protein calsequestrin are expressed at lower levels in atria [21]. The SR Ca^2+^ uptake, the Ca^2+^ content and the cellular Ca^2+^ buffer capacity is higher in atrial compared to ventricular myocytes [22]. Also the contractility between atrial and ventricular cardiomyocytes differs [23]. As nexilin was identified as a protein of the junctional membrane complex (JMC) and loss of nexilin reduced the transversal component of the tubular network [5,6], it is not unlikely that the observed differences in the distribution of nexilin isoforms relate to the absence of the T-tubule network in the mouse atria compared to ventricles. Small mammals in particular (e.g., mice, rats, rabbits) possess only rudimentary T-tubules in atria, if any at all [24,25,26]. In contrast, larger mammals (e.g., sheep, horses, humans) develop well-organized T-tubule networks in atria [27,28]. As for the ventricles, no differences in T-tubule networks were described between small and large species. Thus, one could speculate that truncated nexilin variants preferentially localize to T-tubules, which explains their low abundance in mouse atria. It needs to be further investigated how then a lack of exon 6 and exon 6–8 cluster (which leads to reduced self-interaction) relates to proper function of nexilin in T-tubule networks. At present, T-tubule biogenesis is only at the beginning of understanding. However, it is of great importance to better understand the mechanisms of T-tubule remodeling after heart failure and to identify the key players involved. In a recent work by Caldwell and colleagues, the restoration of atrial T-tubules after heart failure was analyzed in a sheep model. Ultra-structural analysis of the recovered atrial T-tubules revealed disordered morphology (longer and more branched) and also disordered mitochondria. But the newly generated T-tubules were functional in terms of calcium transient [29]. Associated with the T-tubule reformation, increased levels of bridging integrator 1 (BIN1), telethonin (Tcap) and myotubularin (MTM1)—but not JPH2—were observed. Here, the authors assume that MTM1 is one of the main modulators of T-tubule formation in the model used. As mentioned above, there could be differences in the process of T-tubule maturation and remodeling after heart failure between the different species, as there could also be differences in these processes between the respective heart chambers. The potential functions of specific nexilin splice variants in these T-tubule remodeling processes are of great importance.

Another question that remains is: Does the full-length nexilin variant in atria (observed on mRNA level) correspond to the variant which was detected by immunostaining at intercalated discs (Figure 4F,H) and/or to the variant detected in endothelial cells (Figure 4B,C,G,I)? Identification of interaction partners which bind to the region of the exon 6 or of the exon 6–8 cluster might help to unravel the function of full-length nexilin in these areas/cells.

Although the findings of the present study revealed novel nexilin splice variants, there are also limitations that have to be considered. (i) Due to limited access to human heart samples, we were unable to decipher the distribution of nexilin variants in the human atrium vs. ventricle. This is of particular interest as the heart regions of mice and humans show fundamental morphological differences, especially with respect to the atrial/ventricular regions. (ii) When analyzing transcripts with primers targeting internal exons, important information is likely to be missing as this approach is “blind” to the sequence outside the amplicon. Accordingly, the approach presented here may also have missed variants because we decided to analyze only transcripts carrying exon 2 and exon 13/14 (translation start and stop). It is therefore not unlikely that certain transcripts were not detected, as we also obtained PCR products containing exon 3 in epithelial cells (by simply placing the forward primer in exon 3). However, we did not succeed in characterizing these transcripts up- and downstream by running 3′- and 5′-RACE (rapid amplification of cDNA ends). Nevertheless, it can be noted that none of the mature transcripts we observed in epithelial cells (spanning from exon 2 to 13/14) carried exon 3.

## 5. Conclusions

Here, we provide the first detailed description of a variety of alternatively spliced nexilin transcripts in several mouse and human tissues. Our results clearly show a tissue-specific expression pattern of nexilin variants (e.g., kidney, urinary bladder, heart) and a different localization of the variants in the heart (atrium vs. ventricle). These variants also localized differently at the subcellular level (cytosolic vs. membrane-associated). We hope that this will provide a basis for a better understanding of the function of nexilin in areas of the heart and also in tissues other than muscle. Future studies on nexilin expression and function should take into account the different expression patterns of nexilin variants and should provide information on complete transcripts whenever possible.

## Figures and Tables

**Table 1:** Primer used for transcript analysis in human and mouse tissues.

Name	Oligo No.	Sequence (5′–3′)
huNexn-Ex2-fwd	ON979	ATGAATGATATTTCCCAAAAGGC
huNexn-Ex13-rev	ON980	TTAATTCTTACTTTCAATGGTAAG
huNexn-Ex13/14-rev	ON2205	GCTAGTAGTCATCAGTTTCAATGG
huNexn-Ex14-rev (UTR)	ON1956	AGTCAGCATAGTGGCACTCC
huNexn-Ex14-rev	ON2204	GAGGGGAGCTAGTAGTCATCAG
msNexn-Ex2-fwd	ON1014	ATGAATGACGTTTCGCAAAAG
msNexn-Ex2-fwd	ON2196	TACAAGGCTTCAGCCCCAAA
msNexn-Ex3-fwd	ON1705	CAGAGGGCCAGGGAAGAAAG
msNexn-Ex4/5-rev	ON2101	ACTGTCCCTGTTAGCTTTGG
msNexn-Ex5-fwd	ON2059	GGAGAAACACAGACAGGAAGAA
msNexn-Ex5-fwd	ON2061	CGCAGAATTGAGCAGGATCT
msNexn-Ex6-rev	ON1829	CGGACTCGGTTCCCGTATT
msNexn-Ex7-rev	ON2060	TTTGGCAGGTACCACTGTTAT
msNexn-Ex9-fwd	ON1707	TGCTGAAGCAAGGAGAAGCA
msNexn-Ex9-rev	ON2063	GTCTTCTCTCCTTTGCCTTTCT
msNexn-Ex10-rev	ON1765	TGCGTTCTTCCTCTGTTCGT
msNexn-Ex14-rev	ON1015	CTAGTAGTCATCCATTTCAATG
msTtn-fwd	ON1999	GCAAAGCCTCCAATGAGTATGG
msTtn-rev	ON2000	AGGAAGTAATTTACGAACTTTCTTTTCAG
msCdh1-fwd	ON1414	GGACGTCCATGTGTGTGACT
msCdh1-rev	ON1415	GATCAGAATCAGCAGGGCGA
hu/msRN18S-fwd	ON1151	GCAATTATTCCCCATGAACG
hu/msRN18S-rev	ON1152	GGGACTTAATCAACGCAAGC
msNexn∆3 probe (Ex2/4)	P10	FAM-CGCAAAAGGCAGAGATTAAAGAAATGCTT-BHQ1
msNexn∆6 probe (Ex5/7)	P1	FAM-AGAGCGGAGCAGGAAGGAGATGACT-BHQ1
msNexn∆6–8 probe (Ex5/9)	P3	FAM-AGAGCGGAGCAGGTAAATGAGGAGGA-BHQ1

Human: hu, mouse: ms, forward: fwd, reverse: rev, oligonucleotide: ON, untranslated region: UTR, Taqman probe: P, FAM: fluorescein amidite, BHQ1: black hole quencher-1.

## Data Availability

The original contributions presented in the study are included in the article, further inquiries can be directed to the corresponding author.
